# Serotonin Transporter Gene Polymorphisms and Early Parent-Infant Interactions Are Related to Adult Male Heart Rate Response to Female Crying

**DOI:** 10.3389/fphys.2017.00111

**Published:** 2017-02-28

**Authors:** Anna Truzzi, Marc H. Bornstein, Vincenzo P. Senese, Kazuyuki Shinohara, Peipei Setoh, Gianluca Esposito

**Affiliations:** ^1^Department of Psychology and Cognitive Science, University of TrentoRovereto, Italy; ^2^Laboratory for Affiliative Social Behavior, Brain Science Institute, RIKENSaitama, Japan; ^3^Child and Family Research, Eunice Kennedy Shriver National Institute of Child Health and Human DevelopmentBethesda, MD, USA; ^4^Psychometric Laboratory, Department of Psychology, Second University of NaplesNaples, Italy; ^5^Unit of Basic Medical Sciences, Department of Neurobiology and Behavior, Nagasaki UniversityNagasaki, Japan; ^6^Division of Psychology, School of Humanities and Social Sciences, Nanyang Technological UniversitySingapore, Singapore

**Keywords:** parent-infant interaction, serotonin transporter gene, opposite-sex conspecific interaction, gene^*^ environment, physiological responses, social distress

## Abstract

Adults' adaptive interactions with intimate partners enhance well-being. Here we hypothesized that adult males' physiological responses to opposite-sex conspecifics' distress result from an interaction between an environmental factor (early social interaction with caregivers) and a genetic factor (a polymorphism within the promoter region of the serotonin transporter gene, 5-HTTLPR). We assessed heart rate changes in 42 non-married male adults to distress vocalizations (female, infant, and bonobo cries). Males' early interaction with parents was assessed using the Parental Bonding Instrument. Buccal mucosa cell samples were collected to assess their 5-HTTLPR genotype. A significant interaction emerged between early experience and genetic predisposition. Males with a genetic predisposition for higher sensitivity to environmental factors showed atypical physiological responses to adult female cries according to their experienced early maternal parenting. Environmental experiences and genetic characteristics are associated with adult males' physiological responses to socially meaningfully stimuli. Understanding the mechanisms that modulate responses to opposite-sex conspecifics may improve personal well-being and social adaptiveness.

## Introduction

Several early environmental factors greatly influence individuals' long-term development of social behaviors (Sroufe, 2006; Truzzi et al., 2016). Between these factors, social experiences with parents especially contribute to child socio-emotional development, which, in turn, influences the development of adults' relationships with intimate partners (see, e.g., Feeney and Noller, 1990; Simpson, 1990). In the first years of life, the way parents answer infants' interactive behaviors helps to create in individuals' expectancies about social interactions and affects individual development in several domains, especially social abilities, with effects that last into adulthood (Cutrona et al., 1994; Allen et al., 2002; DiTommaso et al., 2003). Prompt and adequate parental responses to infants' needs make infants feel secure toward parents and promote the development of positive expectancies toward others.

Children who experience positive interactions with their parents expect social partners to be available both emotionally and behaviorally and tend to cope well with stressful events, responding to distress with calm and adaptive physiological responses (Sbarra and Hazan, 2008). By contrast, children who do not experience positive interactions with their parents tend to develop maladaptive behavioral and physiological responses toward people and distressing daily problematic events (Sbarra and Hazan, 2008). In turn, higher distress can hinder relationships with intimate partners. Moreover, adults' social interactions with intimate partners are significantly affected by their childhood experiences with the opposite-sex parent. Hazan and Shaver (1987); Apostolidou (2006) found a strong association between males' experience of maternal protection during childhood and their anxiety toward opposite-sex intimate partners during adulthood. Thus, boy infants, who experienced overprotective maternal behaviors, likely suffer higher levels of anxiety in adult relationships with opposite-sex intimate partners. However, the mechanisms which allow early social experience effects to endure into adulthood are poorly known and under-investigated. Here we propose a physiological-mediated mechanism: although physiological reactions may change according to individuals' experience throughout lifetime, early parental behaviors shape the initial development and regulation of individuals' physiological patterns and activations deeply enhancing the probability for us to react to external stimulation in adulthood coherently with early experiences.

At the molecular level specific neurotransmitters, such as serotonin, are involved in interactions with opposite-sex conspecifics. The serotoninergic system is involved in mating (Fisher, 2000). Confronted with a conspecific of the opposite sex, people undergo a sharp decrease in serotonin levels. However, serotonin may not have the same effects on different individuals. Serotonin level is modulated by the serotonin transporter, which depends on the serotonin transporter gene (SLC6A4) genotype. In the general population, the promoter region of the serotonin transporter gene (5-HTTLPR) is a polymorphism found in two distinct forms: the long (L) allele and the short (S) allele, where the less transcriptionally efficient S-allele is associated with lower reuptake of serotonin compared to the more efficient L-allele (Canali and Lesch, 2007). The difference in serotonin reuptake has a deep impact on individual physiology and well-being; indeed, S/S genotype has been found related to a higher probability of developing depression in response to stressful life events (Kendler et al., 2005). Also, different 5-HTTLPR genotypes have been linked to distinct levels of social adaptiveness and have been found to be associated with adult social development. L/L homozygotes enjoy higher social adaptiveness, as evidenced for example by reduced intergroup bias (Cheon et al., 2014), whereas the S/S genotype is linked to more socially maladaptive behaviors. Also, in larger societies with higher democracy levels, when the prevalence of the S allele is greater, people tend to rate the society as less trustworthy (Kong, 2015). Concerning, specifically, the development of interactions with opposite-sex conspecific in adulthood, Caspers et al. (2009) reported that the presence of the S allele in the 5-HTTLPR polymorphism likely is related to poorer bond representations in adulthood.

Both environmental and genetic factors are associated with the development of social abilities and opposite-sex relationships in adulthood, and these associations may be mediated physiologically. To begin to specify and better understand mechanisms underlying these assertions, and point the way to improving adults' interactions with opposite-sex conspecifics, it is important to investigate how gene^*^environment interactions might relate to individuals' physiological responses to mating-relevant stimuli. This study aimed to investigate how early parent-infant interaction and serotonin transporter gene genotype associate with adults' physiological responses to expressions of distress in opposite-sex conspecifics. We hypothesized a gene^*^environment interaction (5-HTTLPR polymorphism genotype^*^parental bonding in childhood) on autonomic nervous system responses. Specifically, considering the evidence about the role of parental behaviors and 5-HTTLPR genotype in influencing adults' social responses, we expected L allele carriers who reported having had better relationships with their parents to show more adaptive reactions to female cries (more calming responses), whereas L carriers who experienced poorer parent-infant relationships to show less adaptive responses (heightened arousal and distress in response to female cries). By contrast, we expected S/S homozygotes to show higher distressing physiological arousal in response to female cries compared to L carriers, independent of reported parent-infant relationship.

## Materials and methods

### Participants

Forty-two non-married male adults (*M* = 24.7 years, *SD* = 5.05) were recruited through a database of volunteers available through web-announcements. Only woman adult cries were available, therefore we only included non-married males to specifically investigate responses to opposite-sex (female) conspecifics' vocalized distress avoiding the effect of a prolonged exposure to an intimate relationship. Written informed consent was obtained from all participants, and the study was conducted in accord with ethical principles stated in the Helsinki declaration. The genetic assessment was conducted on anonymized bio-samples at the University of Nagasaki (Japan), and followed the procedures approved by the IRB guidelines of the University of the Nagasaki Graduate School of Medicine.

### Stimuli

The stimuli were 30 audio clips of distressing vocalizations of 15 s each, belonging to three categories of 10 examples each: female cries, infant cries, and bonobo cries. Cries were chosen because of their evolutionary significance and because they have been found to elicit distress and specific physiological responses in adults (Messina et al., 2016). However, this research specifically aimed to assess physiological responses elicited by opposite-sex conspecific distress, therefore to consider a general effect of cry both infant and female cries were included as stimuli. Also, to test whether the investigated physiological activations were specific to human distress vocalizations or a generalized response to social distress, bonobo cries were included in the stimulus set. Each audio clip was presented following 10 s of silence. There was no significant difference [*F*_(2, 27)_ = 0.28; *ns*] in the mean fundamental frequency of each category of cry: female cries: *f* 0 M = 480 Hz (*f* 0 range = 280–705 Hz); infant cries: *f* 0 M = 482 Hz (*f* 0 range = 334–736 Hz); bonobo cries: *f* 0 M = 384 Hz (*f* 0 range = 355–431 Hz). The audio clips presentation order was randomized three times, creating three presentation sequences. Each participant was assigned one presentation sequence among the three sequences available, so that the presentation order was counterbalanced across participants. Overall, all participants viewed all the video clips. Stimulus sequences were created using open source software Audacity. All stimuli were normalized for intensity (*M* = 85 dB), and the volume was kept constant for all the presentations for all participants.

### Procedure

First, participants completed an online self-report questionnaire to assess their parent-infant history and status. Next, participants' heart rate was recorded throughout the auditory presentation. Participants were seated at about 1 m from the speakers. Before the beginning of the audio sequence, 30 s of heart rate were recorded to assess participants' physiological baseline: participants had been seated throughout electrode positioning, and the baseline was collected in silence and darkness. Finally, a buccal mucosa sample was collected from each participant.

### Parental bonding

The Parental Bonding Instrument (PBI; Parker et al., 1979) is a 50-item self-report questionnaire developed to measure the principal parental dimensions of care and overprotection. Participants filled in two forms, one for maternal, and one for paternal behaviors. Care measures parental attention to needs, and overprotection measures parental protectiveness. Both dimensions, care and overprotection, are measured on continuous scales, where values range from 0 to 3. In our sample, the PBI Cronbach's alphas were for maternal PBI (care α = 0.73; overprotection α = 0.73) and for paternal PBI (care α = 0.65; overprotection α = 0.83). High scores on the care dimension are interpreted as high parental warmth, affection, emotional closeness, and empathy (Arrindell et al., 1998; Dalsant et al., 2015). High scores on the overprotection dimension represent too great parental control, intrusion, and prevention of independent behavior (Arrindell et al., 1998; Rikhye et al., 2008).

### Heart rate

We measured heart rate (HR) using a pulse oximeter (CONTEC CMS60D) placed on participants' left forefinger. The sampling rate of the Oximeter (CONTEC CMS60D) was 64 Hz, and it recorded at a 1 HZ sampling rate (one value per s, where each measure represents the average of the previous 64 samples). Hear Rate activity is under the control of both Automatic Nervous System branches; therefore, to index ANS overall activation participants' heart rate was recorded. A heart rate increase reflects heightened distress, attention, and promptness to action, whereas a heart rate decrease reflects a calming response in reaction to external stimulation (Berntson et al., 1997; Bradley, 2009). A decrease in heart rate may also reflect a decrease of attention; however, this is not inconsistent with a calming response because if, in response to an external distressing stimuli, individuals' attention decreases this is an index of decreased arousal.

### Genetic assessment

DNA extraction and genotyping were conducted by ACGT, Inc. (Wheeling, IL). DNA was extracted from each kit using the Oragene DNA purification reagent as per manufacturer's instructions. DNA concentrations were evaluated using spectroscopy (NanoDrop Technologies, USA). Each DNA sample was polymerase chain reaction (PCR) amplified for the 5HTT repeat region target with the forward primer (50-CCAGCACCTAACCCCTAAT-30) labeled with 6-FAM (6-carboxyfluorescein), and a reverse primer (50-AGGGACTGAGCTGGACAACCAC-30). A PCR reaction of 20 ll consisting of 1.5 ll of genomic DNA from the test sample, PCR buffer, 1 mMeach of forward and reverse primers, 10 mM deoxyribonucleotides, KapaTaq polymerase, and 50 mM MgCl2 was performed. Cycling conditions included an initial 15 min denaturation at 95 _C, and 35 cycles of 94 _C (30 s), 60 _C (60 s), 72 _C (60 s), and a final extension of 72 _C for 10 min. PCR reactions were genotyped with an ABI 3730xl Genetic Analyzer (Applied Biosystems Inc.) and normalized with GeneScan 600 LIZ (Applied Biosystems, Inc.) size standards run on each sample. The genotype data were analyzed using GeneMapper ID (Applied Biosystems, Inc.). To assess the specific effect of the S allele on individual development, participants possessing at least one L allele (L/L or L/S) were classified into a single L carriers group (*N* = 30), and S/S homozygotes were considered as a second group (*N* = 12). S/S homozygotes were compared with L carriers because, S being the less functional allele and given the limited sample size, we were interested in comparing individuals carrying two less functional alleles to other individuals to investigate a stronger effect.

### Analysis

Residuals of the linear model between HR values and HR baseline were calculated. Then, an average level of HR was calculated for each participant in response to each stimulus type (female cries, infant cries, bonobo cries). Outliers, defined as values 2 *SD*s above/below the mean, were replaced with the mean of the distribution. The dependent variable was normally distributed and no transformation was needed (skewness = 0.13). A mixed ANOVA was performed with the calculated HR average as the dependent variable, the cry type (female, infant, bonobo) as a within-subject factor, the 5-HTTLPR polymorphism genotype (L carriers, SS) as a between-subject factor, and the four PBI dimensions (maternal care, paternal care, maternal overprotection, paternal overprotection) as continuous covariates. Regression coefficients were used to analyze the effect of the covariates on the dependent variable; Cohen's *d* was used to evaluate the size of effects.

## Results

### Maternal overprotection

**Figure 1:**
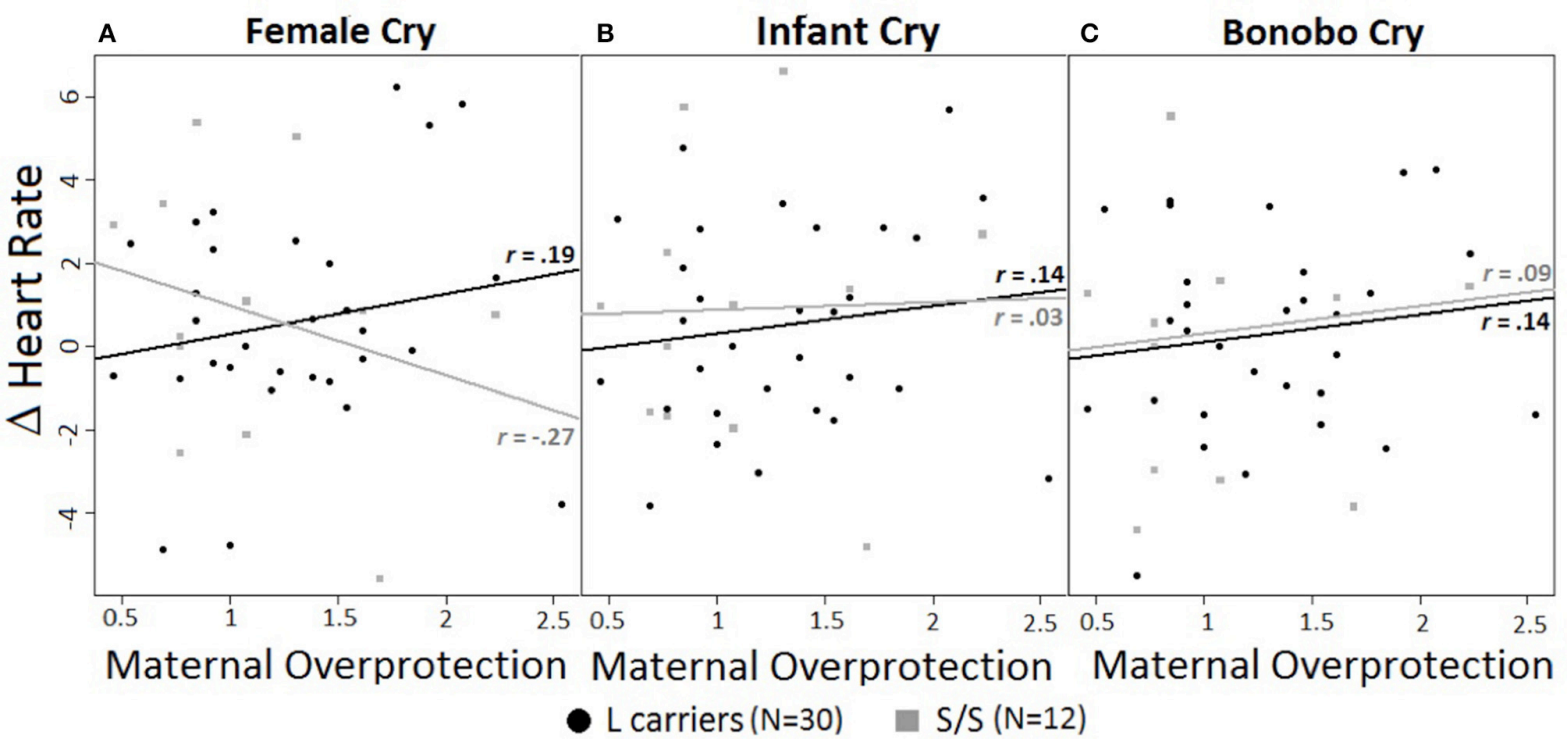
**(A–C)** Correlations between heart rate changes (calculated as difference from baseline) and experienced maternal overprotection in response to different cry types. Maternal overprotection values ranged from 0.46 to 2.54. Black circles = L carriers; gray squares = S/S homozygous. *r*-values represent Pearson correlation. **(A)** HR responses to female cry. Influence of maternal overprotection on HR responses to female cries accordingly to genotype. **(B)** HR response to infant cry. **(C)** HR response to bonobo cry. Lines represent the linear models for L carriers (black) and S/S homozygous (gray).

A significant 3-way interaction of maternal overprotection, genotype, and cry type emerged for HR [*F*_(1, 41)_ = 6.37, *p* < 0.01, *d* = 0.84] (Figures 1A–C). For female cries alone L carriers showed an opposite pattern of activation compared to S/S homozygotes, although the linear regression slopes did not significantly differ. Specifically, for L carriers the higher their reported maternal overprotection, the greater their HR increase, *r* = 0.19, *R*^2^ = 0.04, *p* = 0.33; but for S/S homozygotes the higher their reported maternal overprotection, the greater their HR decrease, *r* = −0.27, *R*^2^ = 0.07, *p* = 0.39 (Figure 1A). In both cases the effect sizes were small. In response to infant and bonobo cries, there were no effects of genotype or maternal overprotection (Figures 1B–C).

### Maternal care and paternal care/overprotection

No significant interactions or main effects for maternal care and genotype and cry type were found on HR. No significant interactions or main effects either for parental care or overprotection and genotype and cry type were found on HR.

## Discussion

Adaptive social interactions between males and females enhance mating likelihood and increase individual and familial well-being. The way individuals respond to socially relevant stimuli in adulthood is affected by environmental factors and by genetic predispositions. Good parent-infant interactions lead to higher levels of adaptive interaction with intimate partners in adulthood, whereas experiences of poorer parental bonding may generate inferior expectations toward social affiliations and, therefore, unwholesome patterns of interaction with opposite-sex partners. Early relationships with opposite-sex parents especially project their influence to adults' interactions to opposite-sex conspecifics (Apostolidou, 2006). At the molecular level, the serotonin neurotransmitter is involved in mating (Fisher, 2000). Because the effect of neurotransmitters depends on the functionality of receptors and transporter proteins on cell surfaces, different genotypes coding for these proteins are thought to directly influence social behavior. Indeed, the presence of a short allele (S) in the functional promoter of the serotonin transporter gene (5-HTTLPR), which reduces both serotonin transporter proteins' transcription and functionality resulting in less efficient serotonin reuptake from the synaptic gap, has been linked to lower levels of social adaptiveness and to poorer expectations of intimate relationships in adulthood (Canali and Lesch, 2007; Caspers et al., 2009; Cheon et al., 2014).

The present study aimed to investigate how early experiences of relationships with biological parents and serotonin transporter gene promoter genotype are associated toadults' physiological responses to distress in opposite-sex conspecifics. Specifically, we expected L carrier individuals who experienced good parenting to show calming responses to female cries, and L carriers who report poorer parenting to show distressing physiological reactions. While we expected S/S homozygotes to show overall higher maladaptive physiological regulation to distressing vocalizations compared to L carriers. In response to female distress vocalizations, differential physiological responses were found in L carriers vs. S/S homozygotes. As expected, among L carriers greater maternal overprotection was associated with increases in heart rate in response to female cries. While, unexpectedly, S/S homozygotes showed an opposite pattern, where the higher the maternal overprotection the greater HR decrease only in response to female cry. This decrease, which underlies a calming response, was unexpected; however, a possible explanation might be found in the evolutionary value of the 5HTTLPR polymorphism. Even if the S allele is less frequent its presence in the population approximates 10%. Such a percentage rules out the possibility for the S allele to be only a casual mutation or a gene flow. Rather, it suggests a balancing evolutionary pressure and the presence of a plastic genotype (Belsky et al., 2009; Esposito et al., 2016) both the L and the S alleles should benefit human beings under different circumstances, highlighting the role of genetic characteristics in moderating individual sensitivity to external environmental factors (Pluess et al., 2010). Individuals' carrying the S allele have been linked to higher stress reactivity and higher amygdala activation in response to stressful stimuli (Hariri et al., 2002; Gotlib et al., 2008). Dobson and Brent (2013) suggested that in a good or favorable environment this hypervigilance involves high resources even to evaluate simple non-dangerous stimuli consuming energies which would better be used in other tasks and leading, as a result, to less adaptive responses to stimuli. However, within a less stable environment more prone to intra-group competition, the hypervigilance that presents in individuals carrying the S allele would be more adaptive as expressed in adaptive responses to distress. Therefore, besides the moderation of 5-HLTTPR genotype on responses to social stimuli and taking into account the competition intrinsic in the mating context, we should also expect an association between the presence of the S allele and specific responses to mating-related stimuli. Consistently with Dobson and Brent (2013), S/S homozygous who experienced low maternal overprotection in childhood showed augmented arousal and promptness to action in response to female cries, but if exposed to a less favorable early environment, such as greater maternal overprotection, they showed diminished arousal and slower readiness to action to female cries. S/S homozygosity seems to be related to adults being less responsive to opposite-sex conspecific distress signaling when they report a less healthy interactive history with their other-sex parent. Different genotypes on the 5-HTTLPR gene appear be associated with sensitivity to early social environmental experiences. Therefore, the same levels of maternal overprotection may have differential effects on development in relation to individual genotypes.

### Limitations

The small sample size is an important limit of this study which reduces the statistical significance of the findings, leading also to weak correlational strength. However, following Cohen's indications, the effect of the interaction between maternal overprotection, genotype and cry type over HR responses is a large effect. Therefore, we feel findings from this research provide initial insight on how the mechanisms underlying opposite-sex conspecific interactions work. To better interpret the meaning of this specific physiological activation future research might measure participants' attitude. Furthermore, it is important to highlight that the present research is correlational in nature, and so not causal. To explore underlying mechanism, a causal study is needed to better clarify the exact roles of genetics and environment on adults' responses during opposite-sex conspecific interactions.

### Conclusions

The interaction between genetic factors and environmental experiences is associated with adult males' physiological reactions to opposite-sex conspecific expressions of distress. It may be that more equanimous responses to stress improve couples' functioning and dyadic well-being. Understanding how sources of adults' social bonds are influenced by genetic predispositions and parent-infant interactions may improve individual well-being and perhaps societal harmony.

## Author contribution

Conceived, designed, and performed the study: AT, MB, PS, GE. Analyzed the data: AT, VS, KS, GE. Interpreted results and wrote the paper: AT, MB, VS, KS, PS, GE.

### Conflict of interest statement

The authors declare that the research was conducted in the absence of any commercial or financial relationships that could be construed as a potential conflict of interest.
